# Contemporary international practice patterns in the prevention, diagnosis, and management of delayed gastric conduit emptying after oesophagectomy: an international survey of oesophagogastric surgeons

**DOI:** 10.1093/dote/doag115

**Published:** 2026-10-20

**Authors:** Jonathan Sivakumar, Mohamed Abulazayem, Michael W Hii, Feras Alnimri, Alex Boddy, Yirupaiahgari K S Viswanath, Carlos Cabalag, David S Liu, Jessie A Elliott, Yogesh Vashist, Cuong P Duong, TUGSS Collaborative Group

**Affiliations:** Department of Surgery, The University of Melbourne, Melbourne, Victoria 3010, Australia; Department of Upper Gastrointestinal Surgery, St Vincent's Hospital Melbourne, 41 Victoria Parade, Fitzroy, Victoria 3065, Australia; Division of Cancer Surgery, Peter MacCallum Cancer Centre, Victorian Comprehensive Cancer Centre, 305 Grattan Street, Melbourne, Victoria 3000, Australia; Imperial College London, Exhibition Road, South Kensington, London SW7 2AZ, UK; Department of Surgery, National Cancer Institute, Cairo University, 1 Kasr Al-Ainy Street, Cairo 11796, Egypt; Department of Surgery, The University of Melbourne, Melbourne, Victoria 3010, Australia; Department of Upper Gastrointestinal Surgery, St Vincent's Hospital Melbourne, 41 Victoria Parade, Fitzroy, Victoria 3065, Australia; Department of Upper Gastrointestinal Surgery, St Vincent's Hospital Melbourne, 41 Victoria Parade, Fitzroy, Victoria 3065, Australia; Department of Surgery, Leicester Royal Infirmary, Infirmary Square, Leicester, Leicestershire LE1 5WW, UK; Department of Upper Gastrointestinal Surgery, The James Cook University Hospital, Marton Road, Middlesbrough, Cleveland TS4 3BW, UK; Division of Surgery, Northern Health, Northern Hospital Epping, 185 Cooper Street, Epping, Victoria 3076, Australia; Division of Cancer Surgery, Peter MacCallum Cancer Centre, Victorian Comprehensive Cancer Centre, 305 Grattan Street, Melbourne, Victoria 3000, Australia; Upper Gastrointestinal Surgery Unit, Austin Hospital, 145 Studley Road, Heidelberg, Victoria 3084, Australia; Victorian Interventional Research and Trials Unit, Department of Surgery, The University of Melbourne, Austin Health, 145 Studley Road, Heidelberg, Victoria 3084, Australia; The Sir Peter MacCallum Department of Oncology, The University of Melbourne, Peter MacCallum Cancer Centre, 305 Grattan Street, Melbourne, Victoria 3000, Australia; National Oesophageal and Gastric Centre, St James's Hospital and Trinity College Dublin, James's Street, Dublin 8, D08 NHY1, Ireland; National Oesophageal and Gastric Centre, St James's Hospital and Trinity College Dublin, James's Street, Dublin 8, D08 NHY1, Ireland; Department of Upper Gastrointestinal Surgery, St Vincent's Hospital Melbourne, 41 Victoria Parade, Fitzroy, Victoria 3065, Australia; Division of Cancer Surgery, Peter MacCallum Cancer Centre, Victorian Comprehensive Cancer Centre, 305 Grattan Street, Melbourne, Victoria 3000, Australia; See Appendix 1 Collaborators

**Keywords:** delayed emptying, gastric conduit, gastric conduit, oesophagectomy, pyloric drainage

## Abstract

Delayed gastric conduit emptying (DGCE) is a common functional complication after oesophagectomy, yet it remains inconsistently defined and managed. Although consensus diagnostic criteria are available, their incorporation into routine audit and the contemporary international patterns of DGCE-related practice have not been characterized. This study aimed to describe current international practice in the prevention, diagnosis and management of DGCE. A cross-sectional online survey was developed and distributed to surgeons performing oesophagectomy internationally through *The Upper Gastrointestinal Surgical Society*. The 55-item instrument captured practice patterns and perceptions relating to DGCE across the perioperative care pathway and during follow-up. Associations were assessed using Pearson chi-square and Fisher’s exact tests. Two hundred and thirty surgeons from 57 countries responded. Fewer than one in five documented early (19.1%) or late (19.6%) DGCE using a standardized audit definition, and incidence was commonly perceived to be less than 10% for both. Prophylactic pyloric intervention was reported by 61.7% of respondents and varied significantly by operative platform, being more frequent among open and hybrid surgeons than minimally invasive or robotic surgeons (*P* = 0.013). Management of established DGCE was highly variable, with conservative and pharmacological measures predominating as first-line therapy and endoscopic pyloric interventions most commonly employed for refractory late DGCE. DGCE practice after oesophagectomy remains highly heterogeneous, shaped by institutional habit as much as by evidence. These findings support wider adoption of standardized DGCE definitions, incorporation of DGCE outcomes into institutional and national registries, and prospective trials comparing prophylactic pyloric strategies.

## INTRODUCTION

Oesophagectomy with gastric conduit reconstruction remains the cornerstone of curative management for resectable oesophageal and oesophagogastric junction cancer.^1^ Advances in multidisciplinary oncological management and structured perioperative care have improved 5-year survival over the past two decades, yet functional complications of reconstruction continue to constrain patient-reported recovery.^2^ Delayed gastric conduit emptying (DGCE) is among the most clinically burdensome of these complications, characterized by impaired transit of gastric contents from the intrathoracic conduit to the duodenum.^3^

Despite increasing recognition of its clinical importance, DGCE remains inconsistently defined and reported.^4^ This heterogeneity has limited meaningful comparison between studies and hindered the development of evidence-based management pathways. A recent expert consensus proposed a standardized diagnostic criteria for DGCE, including clinical and radiological benchmarks distinguishing early DGCE, occurring within 14 days of surgery, from late DGCE thereafter.^5^ Whether these criteria have been adopted in routine clinical audit at an international scale remains unknown.

The prevention and treatment of DGCE also remain areas of ongoing controversy. Available evidence has not established whether prophylactic pyloric intervention confers a clinically meaningful benefit sufficient to outweigh potential risks such as biliary reflux and dumping syndrome.^6^ Postoperative management of established DGCE similarly lacks a consensus treatment algorithm, with supporting evidence drawn predominantly from retrospective, single-center series.^7^ Given the absence of prior studies describing DGCE practice, the present study aims to describe current international practice in DGCE among oesophagogastric surgeons and define priorities for consensus development.

## METHODS

Study design and reporting: This was a cross-sectional, international online survey of oesophago-gastric surgeons regarding clinical practice in relation to oesophagectomy. The study was designed and reported in accordance with the Consensus-Based Checklist for Reporting of Survey Studies.^8^ As this study involved a survey of clinicians, and did not collect patient-level or identifiable participant data, it was acknowledged as exempt from human research ethics approval by the University of Melbourne Human Research Safeguards, Office of Research Ethics and Integrity. The survey was developed and coordinated under the auspices of *The Upper Gastrointestinal Surgical Society (TUGSS)*, which facilitated international dissemination.

### Sampling frame

The target population comprised surgeons independently performing oesophagectomy with gastric conduit reconstruction. Eligible participants were consultant or attending surgeons, or senior fellows with a defined oesophagectomy caseload. No minimum personal case-volume threshold was applied, reflecting the intent to capture a broad spectrum of practice. The sampling frame comprised members of TUGSS who were contacted through membership email lists. Collaborative authorship under the *TUGSS Collaborative Group* was offered to respondents and participating centers.

### Survey development

The English-language questionnaire was developed iteratively by a multidisciplinary steering group of oesophago-gastric surgeons. It was designed to capture current practice patterns and surgeon perspectives on the prevention, diagnosis, and management of DGCE after oesophagectomy (Table S1). The final instrument contained 55 items across 10 thematic domains, hosted on Microsoft Forms (Microsoft, Redmond, WA, USA), and was distributed in May 2026.

### Statistical analysis

Statistical analyses were performed using IBM SPSS Statistics version 31.0 (IBM Corp., Armonk, NY, USA). Categorical variables were summarized as frequencies and percentages. Consistent with previous volume-outcome studies,^9–12^ annual oesophagectomy volume was dichotomized using prespecified thresholds, with low and high surgeon volume defined as 0–5 and >5 cases per year, respectively, and low and high institutional volume defined as <20 and ≥20 cases per year, respectively. Survey responses were analyzed using available-case analysis. Associations between categorical variables were assessed using the Pearson chi-square test. Fisher’s exact test was used when expected cell counts were <5. All tests were two-sided, and a *P*-value of <0.05 was considered statistically significant.

## RESULTS

### Respondent and institutional characteristics

Two hundred and thirty surgeons from 57 countries completed the survey, yielding a response rate of 21.7% (Table 1). The largest national contributions were from India (15.4%), the United Kingdom (11.5%), and Italy (7.0%). The choropleth map in Figure 1 illustrates the international distribution of respondents. Most respondents reported low oesophagectomy volumes, with 53.5% performing 0–5 cases personally per year, and 50.0% working in institutions performing fewer than 20 cases annually. The most frequently reported operative platform was hybrid minimally invasive oesophagectomy, involving either a laparoscopic abdominal phase or a thoracoscopic phase (33.0%), followed by open (27.8%), totally minimally invasive (23.0%), and robotic-assisted approaches (14.8%). Additional operative data of respondents is available in Table S2.

**Table 1:** Respondent demographics and practice characteristics

**Variable**	**n**	**%**
Region of practice		
Asia	95	41.3
Europe	90	39.1
Africa	21	9.1
Oceania	12	5.2
South America	7	3.0
North America	5	2.2
Primary specialty		
Oesophagogastric surgery	85	37.0
General surgery with upper gastrointestinal subspecialty interest	85	37.0
Surgical oncology	39	17.0
Thoracic surgery	9	3.9
Other	12	5.2
Current position		
Consultant/Attending	198	86.1
Fellow	32	13.9
Years independently performing oesophagectomy		
<5 years	109	47.4
5–10 years	56	24.3
11–20 years	42	18.3
>20 years	23	10.0
Personal annual oesophagectomy volume		
0–5	123	53.5
6–15	72	31.3
16–30	23	10.0
>30	12	5.2
Institutional annual oesophagectomy volume		
<20 per year	115	50.0
20–50 per year	82	35.7
51–100 per year	26	11.3
>100 per year	7	3.0
Predominant operative technique		
Ivor Lewis (Two-stage transthoracic)	150	65.2
McKeown (Three-stage cervical anastomosis)	48	20.9
Transhiatal	23	10.0
Left thoracoabdominal	9	3.9
Predominant operative platform/access		
Open	64	27.8
Hybrid minimally invasive: Abdomen (Laparoscopic abdominal and open thoracic phase)	55	23.9
Hybrid minimally invasive: Thorax (Open abdominal and thoracoscopic phase)	21	9.1
Totally minimally invasive	53	23.0
Hybrid robot-assisted	19	8.3
Totally robot-assisted	15	6.5
Primary institution type		
Public hospital (Academic teaching hospital)	192	83.5
Public hospital (Non-academic teaching hospital)	10	4.3
Private hospital	27	11.7
Other	1	0.4

**Fig. 1:**
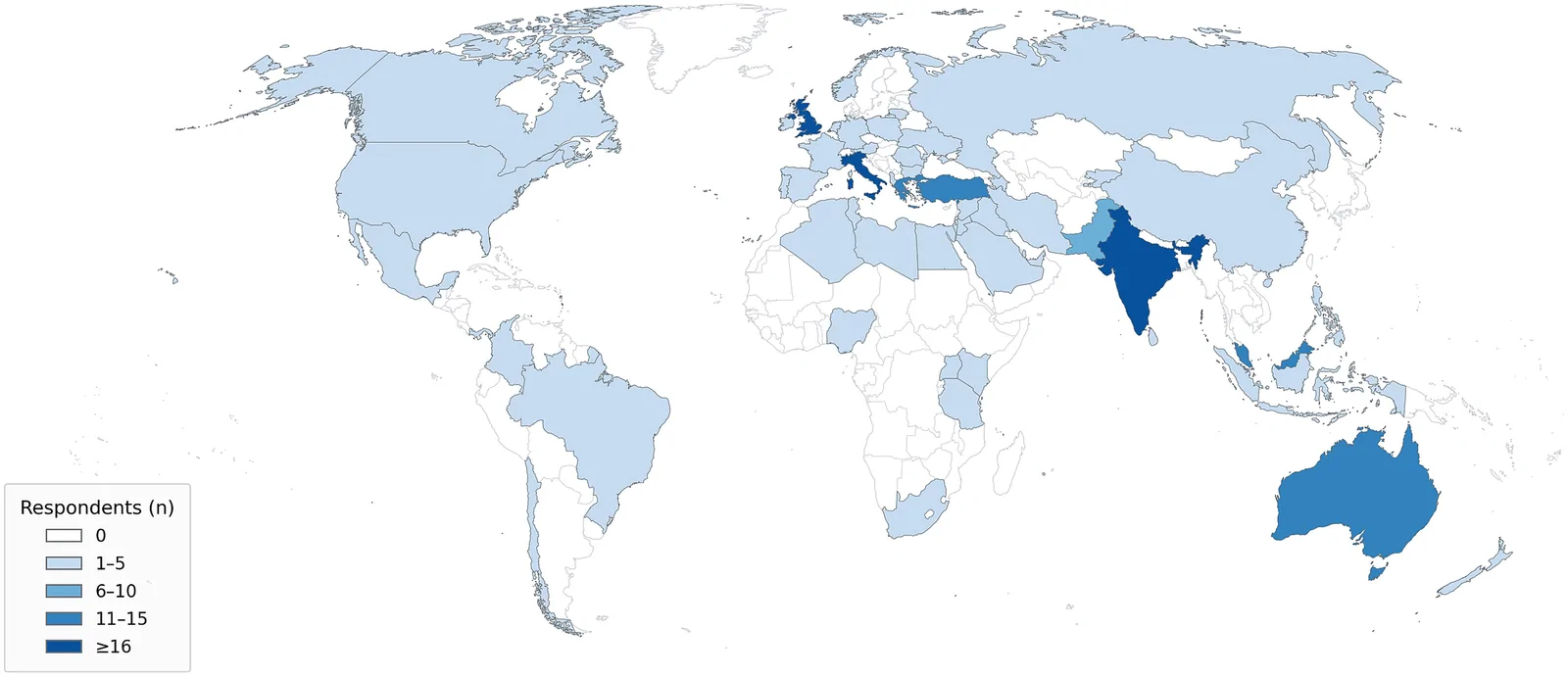
Global distribution of survey respondents by country of practice.

### Nasogastric tube management and early oral intake

Routine nasogastric tube (NGT) insertion at the time of oesophagectomy was reported in 206 of 230 respondents (89.6%). The most frequently cited indications were prevention of conduit distension and aspiration risk (n = 149) and decompression of the anastomosis (n = 123). Criteria for NGT removal were heterogeneous, with the most common being a satisfactory finding on contrast swallow study (35.2%), followed by individual clinical judgment without a fixed criterion (20.9%), volume-based output thresholds (20.9%) and a fixed post-operative day protocol (15.7%). Among respondents applying volume-based criteria, less than 150 mL over 24 hours was the commonest threshold, adopted by 57.4% of volume-based users. As demonstrated in Table 2, NGT removal criteria did not differ significantly by surgeon volume (*P* = 0.908) or institutional volume (*P* = 0.734).

**Table 2:** Nasogastric tube management and oral intake practices by surgeon and institutional case volume

**Variable**		**Annual surgeon volume**		***P*-value**	**Annual institution volume**		***P*-value**
		**≤5 cases**	**>5 cases**		**<20 cases**	**≥20 cases**	
		**n = 123**	**n = 107**		**n = 115**	**n = 115**	
NGT removal criterion, n (%)							
Contrast swallow study		43 (35.0)	38 (35.5)	0.908	40 (34.8)	41 (35.7)	0.734
Individual clinical judgment		27 (22.0)	21 (19.6)		23 (20.0)	25 (21.7)	
Volume-based threshold		24 (19.5)	24 (22.4)		22 (19.1)	26 (22.6)	
Fixed post-operative day protocol		21 (17.1)	15 (14.0)		19 (16.5)	17 (14.8)	
Other		8 (6.5)	9 (8.4)		11 (9.6)	6 (5.2)	
							
Contrast swallow prior to oral intake, n (%)							
Routine		87 (70.7)	51 (47.7)	<0.001	78 (67.8)	60 (52.2)	0.053
Selective only		26 (21.1)	32 (29.9)		23 (20.0)	35 (30.4)	
None		10 (8.1)	24 (22.4)		14 (12.2)	20 (17.4)	
							
Oral free fluid initiation, n(%)							
After contrast swallow (Any POD)		56 (45.5)	34 (31.8)	0.524	49 (42.6)	41 (35.7)	0.154
POD 1		9 (7.3)	11 (10.3)		9 (7.8)	11 (9.6)	
POD 2		12 (9.8)	14 (13.1)		11 (9.6)	15 (13.0)	
POD 3		17 (13.8)	17 (15.9)		14 (12.2)	20 (17.4)	
POD 4		8 (6.5)	14 (13.1)		10 (8.7)	12 (10.4)	
POD 5		13 (10.6)	12 (11.2)		11 (9.6)	14 (12.2)	
Other		8 (6.5)	5 (4.7)		11 (9.6)	2 (1.7)	

*NGT, Nasogastric tube; POD, Post-operative day.*

Contrast swallow study prior to oral intake was performed routinely by 60.0% of respondents. Among routine users, the indication was anastomotic integrity alone (57.2%), conduit emptying alone (13.8%), and both anastomotic integrity and conduit emptying (29.0%). High-volume surgeons were significantly less likely than low-volume surgeons to use routine contrast swallow (47.7% vs. 70.7%; *P* = 0.001). A similar pattern was observed by institutional volume, with surgeons at higher-volume centers performing routine contrast swallow less frequently (52.2% vs. 67.8%; *P* = 0.053). Timing of oral free fluids varied widely with 39.1% of respondents commencing fluids only after a satisfactory contrast swallow irrespective of post-operative day, while the remainder used fixed-day protocols from post-operative day (POD) 1 to POD 5. Fluid initiation timing did not differ significantly by surgeon volume (*P* = 0.524) or institutional volume (*P* = 0.154).

### DGCE definition and estimated incidence

Routine documentation of DGCE in institutional or national audit datasets was inconsistent. Although 57.0% and 55.7% of respondents reported recording early and late DGCE, respectively, as specific complications within their national or institutional databases, fewer than one-in-five used a standardized audit definition, with similar uptake for early DGCE (19.1%) and late DGCE (19.6%). Within this subgroup, exclusive use of the international expert consensus definition was reported by 38.6% of respondents for early DGCE and 57.8% for late DGCE. When asked to indicate the proportion of oesophagectomy patients developing DGCE, the most commonly selected incidence band for early DGCE was 5–10% (31.7%), followed by less than 5% (26.5%). For late DGCE, the most commonly selected incidence band was less than 5% (36.1%), followed by 5-10% (30.0%) (Table S3). The predominant basis for incidence estimates was clinical impression without formal data, informing 42.6% and 43.5% of early and late DGCE estimates, respectively. Formal data sources were used less often, with registry or audit data informing only 16.5–17.8% of estimates. DGCE documentation patterns and perceived incidence did not differ significantly according to surgeon or institutional case volume (Table S4).

### Prophylactic pyloric intervention

Prophylactic pyloric intervention at the time of oesophagectomy was reported by 142 of 230 respondents (61.7%), comprising routine use in 93 and selective use in 49. Among the 142 adopters, pyloroplasty was the most common technique (48.9%), followed by pyloromyotomy (19.9%), digital or finger-fracture dilatation (13.5%), pyloric botulinum toxin injection (8.5%), pyloric balloon dilatation (5.7%), and combined botulinum toxin plus balloon dilatation (2.8%). Among the 88 non-adopters, insufficient evidence of benefit was the most commonly selected reason for omitting prophylactic pyloric intervention (n = 57). The distribution of prophylactic pyloric intervention frequency differed significantly by operative platform (*P* = 0.013; Table 3). Prophylactic pyloric drainage was significantly more common among open and hybrid surgeons than among totally minimally invasive and robotic surgeons (*P* = 0.013). Post-hoc pairwise comparisons with Bonferroni correction, however, indicated that only the difference between hybrid and totally minimally invasive surgery remained statistically significant (*P* = 0.033; Table S5). Among adopters, pyloroplasty was the predominant approach across most platforms, particularly in hybrid surgery (55.6%) and open surgery (48.9%). Robotic surgeons reported a more mixed pattern, with pyloroplasty and pyloromyotomy each used by 25.0% of adopters, and pyloric balloon dilatation and finger-fracture or digital dilatation each used by 18.8% (Fig. 2). Procedure type did not differ significantly by operative platform (*P* = 0.624).

**Table 3:** Prophylactic pyloric intervention frequency and procedure type by operative platform

**Variable**	**Open**	**Hybrid**	**Totally MIS**	**Robotic**	***P*-value**
	**(n = 64)**	**(n = 76)**	**(n = 53)**	**(n = 34)**	
Prophylactic intervention frequency, n (%)					
Routine	31 (48.4)	33 (43.4)	17 (32.1)	11 (32.4)	0.013
Selective	14 (21.9)	22 (28.9)	7 (13.2)	5 (14.7)	
None	19 (29.7)	21 (27.6)	29 (54.7)	18 (52.9)	
					
Procedure type among adopters, n (% of platform adopters)					
Pyloroplasty (Heineke-Mikulicz or similar)	22 (48.9)	30 (55.6)	11 (45.8)	4 (25.0)	0.624
Pyloromyotomy	9 (20.0)	10 (18.5)	5 (20.8)	4 (25.0)	
Pyloric botulinum toxin injection	3 (6.7)	3 (5.6)	4 (16.7)	2 (12.5)	
Pyloric balloon dilatation	1 (2.2)	3 (5.6)	1 (4.2)	3 (18.8)	
Combined pyloric therapy (Botulinum toxin *AND* Balloon dilatation)	2 (4.4)	1 (1.9)	1 (4.2)	0(0.0)	
Finger fracture/Digital dilatation of pylorus	7 (15.6)	7 (13.0)	2 (8.3)	3 (18.8)	
Other	1 (2.2)	0 (0.0)	0 (0.0)	0 (0.0)	

*Data are presented for the four operative platforms. The overall P-value represents Pearson’s chi-square test across the four platforms. Following the omnibus test for prophylactic intervention frequency, post-hoc pairwise chi-square comparisons were performed with Bonferroni correction for six comparisons. Only hybrid versus totally MIS remained statistically significant after correction (Bonferroni-adjusted P = 0.033). Three respondents did not report their operative platform, so column totals sum to 227 rather than the full cohort of 230. MIS, Minimally invasive surgery.*

**Fig. 2:**
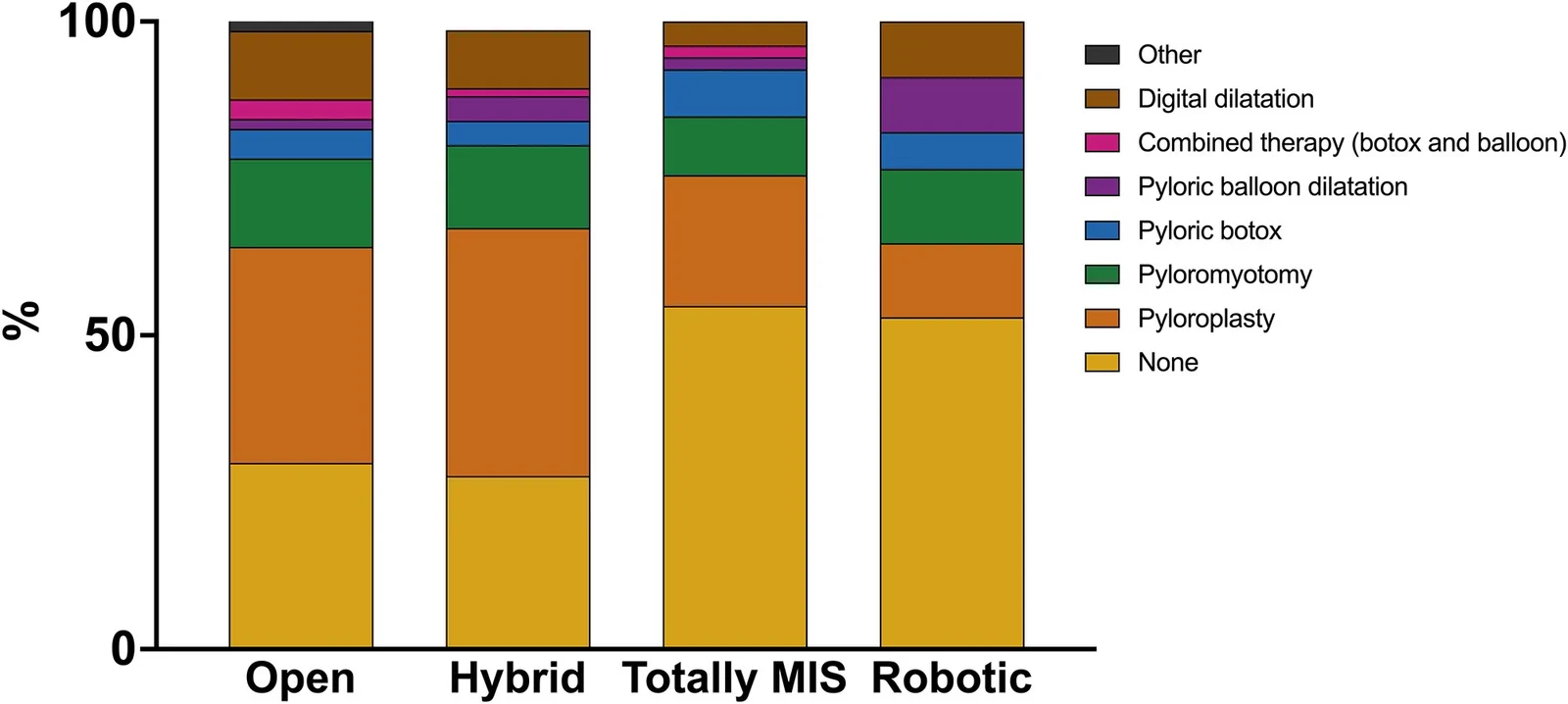
Prophylactic pyloric intervention strategy by operative platform.

### Management of Established DGCE

Surgeons’ management strategies for established DGCE were captured using a multiple-response item, where individual interventions were not mutually exclusive. For early DGCE, first-line management was predominantly conservative and pharmacological. Prokinetic agents were the most frequently employed initial measure (72.6%), followed by continued NGT drainage (63.0%), nil by mouth with parenteral or enteral support (42.6%), and then endoscopic assessment (41.7%). For late DGCE refractory to conservative measures, the most commonly used treatments were endoscopic pyloric balloon dilatation (45.2%), combined pyloric botulinum toxin injection plus balloon dilatation (26.1%), and botulinum toxin injection alone (17.4%) (Fig. 3). Where balloon dilatation was used, a 16–20 mm balloon was most commonly selected; and where botulinum toxin was used, 100 units was the most common dose. Perceived durability of a single endoscopic pyloric intervention was modest, with wide variation in reported response rates and most respondents estimating sustained resolution in no more than half of DGCE cases (Table S6).

**Fig. 3:**
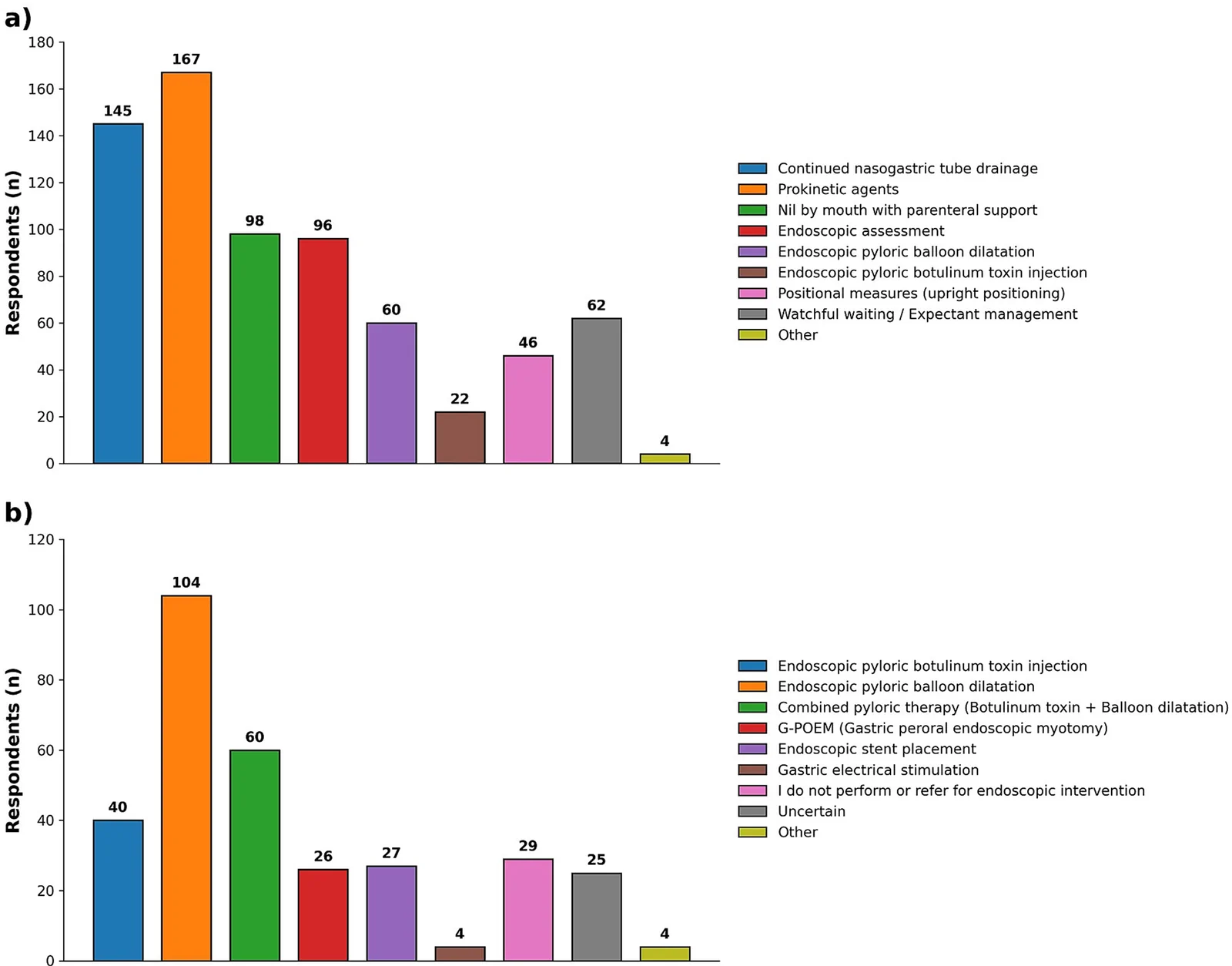
Management of early and late DGCE in the absence of surgically modifiable anatomical pathology, (a) Surgeon-reported first-line management strategies for early DGCE in the absence of anatomical or mechanical obstruction. (b) Surgeon-reported interventions for late DGCE refractory to conservative measures. Bars indicate respondent counts, where multiple selections were permitted.

### Barriers to optimal DGCE management and research priorities

Barriers to optimal DGCE management were assessed using a multiple-response item, and several interrelated themes were evident (Table S7). The most prominent was the absence of a standardized definition for DGCE (61.7%), followed closely by the paucity of high-quality randomized evidence to guide treatment (54.8%). More than one-third cited the lack of DGCE-specific clinical practice guidelines (87/230, 37.8%). These themes were reinforced by the 97 substantive free-text responses describing the single most important unanswered research question. The dominant priorities were the need for adequately powered randomized trials of prophylactic strategies for DGCE, and the development of an operationalizable consensus definition (Table S8).

## DISCUSSION

This international survey demonstrates substantial heterogeneity in DGCE practice after oesophagectomy. Two key findings are particularly important. First, fewer than one-in-five respondents documented early or late DGCE using a standardized audit definition, despite availability of international consensus criteria. Second, prophylactic pyloric intervention remains deeply divided, with similar proportions of surgeons reporting routine use, selective use or complete omission.

The low uptake of standardized DGCE definitions is clinically important. The international Delphi consensus proposed a diagnostic criteria for early DGCE, based on NGT output or radiographic conduit dilatation, and for late DGCE, based on symptom burden combined with objective delayed contrast passage.^5^ These criteria were designed to address the longstanding problem of inconsistent DGCE reporting,^4^ which has limited the interpretability of the literature, in which reported incidences have ranged from 2 to 47%.^13^^,^^14^ Contemporary cohorts applying the consensus definition report early DGCE of 20.0–27.7% and late DGCE of 12.0–30.3%.^15–18^ The relatively low incidences estimated by surgeons in the present survey, largely perceived as less than 10% for both early and late DGCE, highlights the variability in identification and reporting across centers.

This may reflect a combination of heterogeneous diagnostic definitions, variable surveillance intensity, and limited awareness of DGCE in centers that are not structured to systematically detect it, leaving many cases unaccounted for when not actively audited. The international consensus criteria, although valuable for research standardization, may discriminate poorly between patients who ultimately require pyloric intervention when applied in isolation. In a previous single-center comparison, consensus-based thresholds for NGT output and radiographic conduit dilatation identified a patient cohort with a sensitivity and specificity of only 37.9% and 87.3% for predicting the need for subsequent endoscopic dilatation.^15^ A further consideration is that the consensus definition does not always align with real-world practice, where endoscopy is frequently employed as a first-line investigation for suspected DGCE, as also observed in the present study, valued in part because it confers therapeutic, and not merely diagnostic, benefit.^19^^,^^20^ This mismatch between definitions designed for comparability across studies and the pragmatic demands of day-to-day management likely contributes to the slow translation of consensus criteria into routine audit.

Perioperative conduit management was similarly inconsistent. Routine NGT insertion was almost universal, though criteria for removal varied widely. The value of decompression was reinforced by the Nordic kiNETiC trial, in which omission of the nasogastric tube could not be shown non-inferior to 5 days of decompression, and was associated with a higher rate of anastomotic leak.^21^ The *Enhanced Recovery After Surgery Society* likewise recommends nasogastric drainage at resection, with early removal around the second postoperative day when clinically appropriate,^22^ although evidence-based criteria for NGT removal remain an important research priority given the wide variation in current practice.^23^

Contrast swallow practice was also divergent. Approximately half of respondents reported routine use, mainly to assess anastomotic integrity, while high-volume surgeons and institutions used them less often. This is difficult to reconcile with the limited sensitivity of routine contrast studies for detecting anastomotic leak (44%),^24^ and in many series clinically significant leaks manifest before scheduled imaging while carrying a risk of aspiration.^25^^,^^26^ The *Association of Upper Gastrointestinal Surgery of Great Britain and Ireland* also recommend commencing clear oral fluids in the immediate postoperative period, suggesting that doing so doesn’t increase pulmonary complications.^23^ These data and recommendations contrast with the patterns observed in this survey, in which nasogastric and contrast swallow protocols appear to be sustained by local habit and perceived safety margins rather than by high-quality evidence.

Prophylactic pyloric intervention remains a major area of clinical uncertainty. Approximately 62% of surgeons reported using some form of prophylactic drainage, most commonly pyloroplasty or pyloromyotomy. Uptake varied by operative platform with numerically greater use among open and hybrid surgeons than among totally minimally invasive or robotic surgeons. Post-hoc pairwise comparisons with Bonferroni correction reached significance only for hybrid versus totally minimally invasive surgery (p = 0.033), likely due to limited power for individual comparisons after correction rather than a true difference between open and hybrid practice. The observed overall pattern, however, may reflect differences in technical feasibility and reluctance to add operative time or complexity during minimally invasive learning curves. Open or hybrid approaches may render surgical drainage more straightforward, with limited incremental operative burden.^27^ The available evidence on this has also not established whether routine prophylactic drainage confers a consistent clinical benefit. Historical meta-analyses pooled heterogeneous eras, definitions, and techniques, limiting their interpretability.^13^ While some suggested pyloric drainage reduced early DGCE and shortened gastric emptying times, they showed little impact on pulmonary morbidity, anastomotic leak or length-of-stay.^3^^,^^28^ Given their heterogeneity, each pyloric drainage technique warrants separate appraisal rather than aggregation into a single pooled estimate, as has been done in previous meta-analyses. Recent randomized evidence supports this distinction with pyloroplasty showing improved gastric emptying and reduced composite of pneumonia or anastomotic leak,^29^ whereas pyloric botulinum toxin injection has consistently failed to demonstrate any comparable benefit.^30^ Prophylactic endoscopic pyloric balloon dilatation, by contrast, remains supported only by case-series data,^31^^,^^32^ with comparative randomized evidence presently lacking. Two randomized controlled trials are now underway to address this knowledge gap. The PROPPER and WIDE trials aim to evaluate the prophylactic effect of 20 mm and 30 mm balloon dilatation, respectively, in reducing DGCE in contemporary cohorts.^33^^,^^34^ Evidence for pyloromyotomy is also limited, with contemporary comparative evidence derived from a single observational study and no clear reduction in DGCE compared with no pyloric intervention.^35^ These data highlight that the evidence base is procedure-specific, and the benefits demonstrated with pyloroplasty should not be extrapolated to other forms of pyloric drainage.

This is the first international survey focused on DGCE practice after oesophagectomy, with a rigorously developed questionnaire covering the full care pathway. Several limitations should also be acknowledged. First, the data are self-reported and may reflect intended rather than observed practice, which is an inherent bias of survey-based research. Second, the response rate raises the possibility of selection and non-response bias, with surgeons more engaged in DGCE management potentially over-represented. Third, the predominance of lower-volume surgeons may have influenced reported practice patterns and limits interpretation of these findings as an expert consensus from high-volume centers. This distribution does, however, reflect real-world oesophagectomy practice in less-centralized health systems, where most hospitals perform relatively few oesophagectomies annually.^36–38^ Despite these limitations, this study identifies key priorities for future research.

## CONCLUSION

This international survey demonstrates marked heterogeneity in DGCE prevention, diagnosis and management after oesophagectomy. Standardized audit definitions remain uncommon, incidence estimates often rely on clinical impression, and prophylactic pyloric intervention varies by operative platform. These findings support registry-based DGCE capture and prospective trials comparing pyloric strategies.

## Supplementary Material

Supplementary_Material_doag115

## Notes

Specific author contributions: Jonathan Sivakumar (Conceptualization, Data curation, Formal analysis, Investigation, Methodology, Project administration, Resources, Software, Supervision, Validation, Visualization, Writing—original draft, Writing—review & editing), Mohamed Abulazayem (Investigation, Project administration, Resources, Writing—review & editing), Michael W Hii (Methodology, Project administration, Software, Supervision, Validation, Visualization, Writing—review & editing), Feras Alnimri (Conceptualization, Data curation, Formal analysis, Investigation, Resources, Software, Validation, Writing—review & editing), Alex Boddy (Methodology, Project administration, Resources, Validation, Writing—review & editing), Yirupaiahgari KS Viswanath (Project administration, Writing—original draft), Carlos Suhady Cabalag (Methodology, Project administration, Writing—review & editing), Jessie A Elliott (Writing—review & editing), Yogesh Kumar Vashisht (Writing—review & editing), TUGSS Collaborative Group (Data curation).

Financial support: Jonathan Sivakumar is a recipient of and supported by The Alan and Kate Gibson Research Fellowship.
