# Supplementary material for: Contemporary international practice patterns in the prevention, diagnosis, and management of delayed gastric conduit emptying after oesophagectomy: an international survey of oesophagogastric surgeons
Source: Dis Esophagus. 2026 Oct 20;39(5):doag115. doi: 10.1093/dote/doag115 (PMC13682582; doi:10.1093/dote/doag115)
Supplement: Supplementary file 1 [file supplementary_material_doag115.docx]

**SUPPLEMENTARY MATERIAL**

**Supplementary Appendixes**

[Appendix 1. Members of the *TUGSS Publishing Committee* and collaborating investigators	2](#_Toc239711169)

**Supplementary Material**

[Electronic Supplementary Material - Table S1. Structured survey questionnaire.	9](#_Toc239711170)

[Electronic Supplementary Material - Table S2. Operative practice characteristics of respondents.	11](#_Toc239711171)

[Electronic Supplementary Material - Table S3. Estimated incidence of early and late DGCE after oesophagectomy, as reported by survey respondents.	12](#_Toc239711172)

[Electronic Supplementary Material - Table S4. Documentation and perceived incidence of DGCE by surgeon and institutional case volume.	13](#_Toc239711173)

[Electronic Supplementary Material - Table S5. Post-hoc pairwise comparisons of prophylactic pyloric intervention by operative platform, with Bonferroni correction	14](#_Toc239711174)

[Electronic Supplementary Material - Table S6. Surgeon-reported likelihood of sustained DGCE resolution after a single endoscopic pyloric intervention.	15](#_Toc239711175)

[Electronic Supplementary Material - Table S7. Surgeon-reported barriers to optimal DGCE management.	16](#_Toc239711176)

[Electronic Supplementary Material - Table S8. Thematic analysis of surgeon-nominated research priorities for delayed gastric conduit emptying.	17](#_Toc239711177)

# **Appendix 1. Members of the *TUGSS Publishing Committee* and collaborating investigators.**

| **Name** | **Institution** | **Country** |
| --- | --- | --- |
| Abhijit Powar | Aster Aadhar Hospital | India |
| Ademola Adeyeye | Afe Babalola University | Nigeria |
| Aggelos Laliotis | Venizeleio General Hospital | Greece |
| Agron Dogjani | University of Medicine of Tirana | Albania |
| Agustin Rodriguez Gonzalez | Hospital de Clínicas | Paraguay |
| Ahmed Abdelsamad | University of Witten/Herdecke | Germany |
| Ahmed Bahgat Soliman | The Princess Alexandra Hospital NHS Trust | United Kingdom |
| Ahmed Mohamed Suliman | Hamad Medical Corporation | Qatar |
| Ahmed Siddique Ammar | University of Lahore Hospital | Pakistan |
| Ajay Sud | Bradford Royal Infirmary | United Kingdom |
| Ajaz Ahmad Rather | Sher-e-Kashmir Institute of Medical Sciences | India |
| Alessia Fassari | Centre Hospitalier Universitaire de Rennes | France |
| Alessio Giordano | Careggi University Hospital | Italy |
| Alexander L. Ayzengart | Renown Health | United States |
| Ali Guner | Karadeniz Technical University | Turkiye |
| Almu'Atasim Khamees | King Hussein Cancer Center/Jordanian Ministry of Health | Jordan |
| Amit Gupta | All India Institute of Medical Sciences | India |
| Ana-Maria Musina | Grigore T Popa University of Medicine and Pharmacy | Romania |
| Andrea Balla | Hospital Universitario Virgen Macarena | Spain |
| Andrew Hardley | Fiona Stanley Hospital | Australia |
| Andrey Ryabov | Hertsen Moscow Oncology Research Institute | Russian Federation |
| Aneta Obcowska-Hamerska | Medical University of Warsaw | Poland |
| Anis Hasnaoui | Menzel Bourguiba Hospital | Tunisia |
| Anouar Remini | University Hospital of Oran | Algeria |
| Antonio Castaldi | CHU Nîmes | France |
| Appou Tamijmarane | Fakeeh University Hospital | United Arab Emirates |
| Arda Isik | Istanbul Medeniyet University | Turkiye |
| Arif Zuhan | Mataram University | Indonesia |
| Aristotelis Kechagias | Metropolitan General Hospital | Greece |
| Arshad Rashid | SMHS Hospital | India |
| Ashraf Bakri | Latakia University Hospital | Syrian Arab Republic |
| Ashraf Tokhi | St Vincent's Hospital Melbourne | Australia |
| Asma Sghaier | Hôpital Farhat Hached | Tunisia |
| Azimov Elman | Azerbaijan Medical University | Azerbaijan |
| Azize Saroglu | Trakia Hospital | Bulgaria |
| Balakrishnan Selvakumar | All India Institute of Medical Sciences | India |
| Bakyt Toktogaziev | Kyrgyz State Medical Academy | Kyrgyz Republic |
| Bharath Shiva | Netaji Subhash Chandra Bose Medical College Jabalpur Madhya Pradesh | India |
| Bhushan Kumar Yadab | Osh State University | Kyrgyz Republic |
| Bienfait Mumbere Vahwere | Kampala International University | Uganda |
| Bouzid Ahmed | Mahmoud Matri's Hospital | Tunisia |
| Bruno Zilberstein | São Paulo University | Brazil |
| Burak Güney | LIV Hospital Vadistanbul | Turkiye |
| Carlos García Carrasco | Hospital San Borja Arriaran | Chile |
| Celso Roberto Passeri | Hospital Amaral Carvalho | Brazil |
| Chek Tog | Northern Hospital | Australia |
| Christopher John Peters | Imperial College London | United Kingdom |
| Clifford Caruana | Mater dei Hospital | Malta |
| Daniel M Felsenreich | Medical University of Vienna | Austria |
| David James Robert Spence | Belfast City Hospital | United Kingdom |
| Dhiraj Sah | Osh State University International Medical Faculty | Kyrgyz Republic |
| Dhouioui Khaireddine | CHU Farhat Hached | Tunisia |
| Dimitrios Schizas | National and Kapodistrian University of Athens | Greece |
| Duminda Subasinghe | The National Hospital of Sri Lanka | Sri Lanka |
| Efstratia Baili | Hull University Teaching Hospital NHS Trust | United Kingdom |
| Elbashir Osman | University of Pretoria | South Africa |
| Eleftherios Spartalis | REA Maternity Hospital | Greece |
| Erdinc Kamer | University of Health Sciences Turkey | Turkiye |
| Erik Efraín Sosa Durán | Hospital Juárez de México | Mexico |
| Ernest Amos Siwo | Kenyatta University Teaching Referral and Research Hospital | Kenya |
| Ernest Ong Cun Wang | Sarawak General Hospital | Malaysia |
| Evelyn Astrid Dorado Alban | Christus Sinergia Farallones Clinic | Colombia |
| Evgeni Dimitrov | University Hospital Stara Zagora | Bulgaria |
| Evgeniy Drozdov | Siberian State Medical University | Russian Federation |
| Fabio Massimo Oddi | Tor Vergata University | Italy |
| Francisco Tustumi | Einstein Hospital Israelita | Brazil |
| Gennaro Martines | Azienda Ospedaliero Universitaria Policlinico Bari | Italy |
| Giovanna Pavone | University of Foggia | Italy |
| Giovanni Cesana | Policlinico San Marco | Italy |
| Giulio Argenio | AORN San Pio | Italy |
| Giuseppe Brisinda | Fondazione Policlinico Universitario A Gemelli IRCCS | Italy |
| Giuseppe Currò | University Hospital of Catanzaro | Italy |
| Giuseppe Sena | AOU Renato Dulbecco | Italy |
| Gul Sharif | Lady Reading Hospital | Pakistan |
| Hafsa Younus | University College London Hospitals NHS Foundation Trust | United Kingdom |
| Hai T Bui | Western Health | Australia |
| Hans Alexander Mahendran | Hospital Sultanah Aminah | Malaysia |
| Haris Ali Khan | University Hospital Coventry | United Kingdom |
| Harish Neelamraju Lakshmi | Continental Hospitals | India |
| Hassan Ahmed | Patel Hospital | Pakistan |
| Hazaratahiah Nadendla | Apollo Hospitals | India |
| Helio dos Santos da Costa Oliveira | Xi'an Jiaotong University | Timor-Leste |
| Hitesh Sarda | Dr Ram Manohar Lohia Institute of Medical Sciences | India |
| Hoda Salem Alhaddad | Geryan Central Hospital | Libya |
| Hrishikesh Salgaonkar | Fortis Hospital Mulund | India |
| Iea Al-Abbasi | North Devon District Hospital | United Kingdom |
| J K A Jameel | Apollo Hospitals | India |
| J Robert O'Neill | Cambridge Oesophagogastric Centre | United Kingdom |
| Jamal Ahmed Mohamoud | Makerere University | Uganda |
| James Byrne | University Hospital Southampton NHS Foundation Trust | United Kingdom |
| Jan Andrew Bueno | University of Santo Tomas Hospital | Philippines |
| Johnson Mariaantony | Government Tirunelveli Medical College | India |
| Julio Galindo Alvarez | Hospital Universitario Ramón y Cajal | Spain |
| Kaushik Bhattacharya | Mata Gujri Memorial Medical College and LSK Hospital | India |
| Kelvin Voon | Sunway Medical Centre Penang | Malaysia |
| Khalid Bhatti | Royal Marsden Hospital | United Kingdom |
| Kiselev Nikolay | Nizhny Novgorod Regional Clinical Oncological Dispensary | Russian Federation |
| Konstantinos Saliaris | National and Kapodistrian University of Athens | Greece |
| Laurent Genser | Sorbonne Université, Pitié Salpêtrière University Hospital | France |
| Lokesh Agarwal | All India Institute of Medical Sciences | India |
| Lovenish Bains | Maulana Azad Medical College | India |
| Luigi Bonavina | University of Calabria | Italy |
| Luigi Eduardo Conte | Università di Roma Tor Vergata | Italy |
| Luigi Marano | Academy of Applied Medical and Social Sciences | Poland |
| Maciej Walędziak | Military Institute of Medicine | Poland |
| Maham Arshad | Shaukat Khanum Memorial Cancer Hospital and Research Centre | Pakistan |
| Manjunath Siddaiah-Subramanya | Bankstown-Lidcombe Hospital | Australia |
| Manojit Sarkar | Maharaja Jitendra Narayan Medical College and Hospital | India |
| Manuela Mastronardi | University of Trieste | Italy |
| Maria Papadoliopoulou | Attikon University Hospital | Greece |
| Maria Sotiropoulou | Evaggelismos General Hospital | Greece |
| Marta David Sousa | Champalimaud Foundation | Portugal |
| Martynas Luksta | Vilnius University | Lithuania |
| Mary Ann Johnson | St Vincent's Hospital Melbourne | Australia |
| Masoud Rezvani | Inova Fairoaks Hospital | United States |
| Mauricio Zuluaga Zuluaga | Universidad del Valle | Colombia |
| Mauro Podda | University of Cagliari | Italy |
| Mehmet Celal Kızılkaya | Memorial Bahçelievler Hospital | Turkiye |
| Merve Tokocin | Bagcilar Training and Research Hospital | Turkiye |
| Mezhuneituo Raleng | Supercare Hospital | India |
| Michael Devadas | University of Sydney | Australia |
| Michael El-Boghdady | The Royal Surrey County Hospital | United Kingdom |
| Michail Vailas | Laiko General Hospital | Greece |
| Miljana Vladimirov | University Hospital OWL - Campus Lippe | Germany |
| Moataz M Bashah | Weill Cornell Medicine - Qatar & Hamad Medical Corporation | Qatar |
| Mohamed Arif bin Hameed Sultan | Universiti Malaysia Sabah | Malaysia |
| Mohamed Mosaad Mohamed Kandel | Port Said University | Egypt |
| Mohamed Shafi bin Mahboob Ali | Ministry of Higher Education | Malaysia |
| Mohammed Ahmed Omar | Qena University | Egypt |
| Mohammed Al-Rashedy | Warrington and Halton Teaching Hospitals NHS Foundation Trust | United Kingdom |
| Mohd Riyaz Lattoo | Sher-i-Kashmir Institute of Medical Sciences | India |
| Mohie El-Din Mostafa Madany | Aswan University | Egypt |
| Monika Gupta | Manipal Hospital | India |
| Morris Muhinga | Nairobi Hospital | Kenya |
| Muhammad Haekal Khazalle | National Cancer Institute Putrajaya | Malaysia |
| Muhammad Umar Younis | Mediclinic City Hospital | United Arab Emirates |
| Munir Tarazi | Imperial College London | United Kingdom |
| Mustafa Yener Uzunoglu | Bursa City Hospital | Turkiye |
| Muzafferv Önder Öner | Nisantasi University | Turkiye |
| Nader M Hanna | Southlake Health | Canada |
| Nasser Amjad | International Islamic University | Malaysia |
| Naveen Kumar | All India Institute Of Medical Sciences Delhi | India |
| Nik Ritza Kosai | Universiti Kebangsaan Malaysia | Malaysia |
| Nissar Hamdani | Government Medical College | India |
| Noushif Medappil | Meitra Hospital | India |
| Nur Alkrinawi | Soroka University Medical Center | Israel |
| Nuriddin Abdulkhakimov | P. Hertsen Moscow Oncology Research Institute | Russian Federation |
| Nuru Bayramov | Azerbaijan Medical University | Azerbaijan |
| Olga Tucker | University Hospitals Birmingham | United Kingdom |
| Orestis Ioannidis | Aristotle University of Thessaloniki | Greece |
| Paolo Bernante | University of Bologna | Italy |
| Prabhu Nesargikar | HCG Hospital | India |
| Qasim Mohammed al Jameel | Al Sader Medical City | Iraq |
| Radha Raman Mondal | Medical College Kolkata | India |
| Rahul Gupta | Synergy Institute of Medical Sciences | India |
| Rajesh Gianchandani-Moorjani | Hospital Universitario Nuestra Señora de Candelaria | Spain |
| Raju Pareek | University Hospital Wishaw | United Kingdom |
| Ramin Khatibsemnani | Islamic Azad University | Iran |
| Reinaldo Isaacs Beron | Caja de Seguro Social | Panama |
| Renol M Koshy | James Cook University Hospital | United Kingdom |
| Ritesh Patel | Te Whatu Ora Hauora O Toi Tauranga Public Hospital | New Zealand |
| Roy Jaap Jan Verhage | University Medical Center Groningen | Netherlands |
| Salvador Morales-Conde | Hospital Universitario Virgen Macarena | Spain |
| Salvatore Buscemi | ARNAS Civico | Italy |
| Samira Sinacer | Sétif University Hospital Center | Algeria |
| Saurabh Singhal | Aakash Healthcare Superspeciality Hospital | India |
| Sekkapan Thannimalai Sambanthan | Hospital Sultanah Aminah | Malaysia |
| Selmy S Awad | Mansoura University Hospital | Egypt |
| Sercan Yuksel | Başakşehir Çam and Sakura City Hospital | Turkiye |
| Seref Oray | Training and Research Hospital | Turkiye |
| Serge Chooklin | Saint Paraskeva Medical Center | Ukraine |
| Serhat Meriç | Istanbul Aydın University | Türkiye |
| Shaban Mustafa Yuruk | Trakia Hospital | Bulgaria |
| Shahab Shahabi | Iran University of Medical Sciences | Iran |
| Shankar Subbarayan | KAP Viswanatham Government Medical College | India |
| Sharfuddin Chowdhury | Mater Misericordiae University Hospital | Saudi Arabia |
| Sherif Hamed | Nottingham University Hospital Trust | United Kingdom |
| Shree Ram Prasad Chaurasiya | Osh State University | Kyrgyz Republic |
| Simon Msika | Université de Paris APHP | France |
| Sivakumar Gananadha | Canberra Hospital | Australia |
| Sjaak Pouwels | Bielefeld University Medical School | Germany |
| Sofyan Abu Freih | Soroka University Medical Center | Israel |
| Sonam Gupta | Medanta Superspeciality Hospital | India |
| Soumil Jitendra Vyas | Sir Hurkisondas Nurrotumdas Reliance Foundation Hospital and Research Centre | India |
| Stanley Ukadike Okugbo | University of Benin | Nigeria |
| Stefan Morarasu | Grigore T Popa University of Medicine and Pharmacy | Romania |
| Suhaib Ahmad | Health Education and Improvement Wales | United Kingdom |
| Suhail Khuroo | Shri Mata Vaishno Devi Narayana Superspeciality Hospital | India |
| Suvendu Maji | Institute of Post Graduate Medical Education & Research | India |
| Syed Osama Zohaib Ullah | Homerton Hospital | Pakistan |
| Tania Triantafyllou | National and Kapodistrian University of Athens | Greece |
| Tanvir Hossain | Southampton General Hospital | United Kingdom |
| Taryel Omarov | Azerbaijan Medical University | Azerbaijan |
| Tevfik Kıvılcım Uprak | Marmara University | Turkiye |
| Theevashini Krishnasamy | Hospital Al-Sultan Abdullah | Malaysia |
| Tom Mala | Oslo University Hospital | Norway |
| Ufuk Memiş | Erzincan University | Turkiye |
| Umesh Jayarajah | University of Colombo | Sri Lanka |
| Vasileios Charalampakis | Henry Dunant Hospital Center | Greece |
| Venu Bhargava Mulpuri | ESIC Medical College and Hospital | India |
| Victor Turrado-Rodriguez | Hospital Clínic Barcelona | Spain |
| Waleed Mabood | Peshawar Medical College | Pakistan |
| William Malinda Lutege | Muhimbili National Hospital | Tanzania |
| Xuefeng Leng | Sichuan Cancer Hospital & Institute | China |
| Yan Mei Goh | Royal Liverpool Hospital | United Kingdom |
| Yasin Kara | Kanuni Sultan Süleyman Training and Research Hospital | Turkiye |
| Yehonatan Nevo | Sheba Medical Center | Israel |
| Yuen Hua Loh | University Hospitals of Derby and Burton NHS Foundation Trust | United Kingdom |
| Zied Hadrich | Mongi Slim LaMarsa University Hospital | Tunisia |
| Zubair Shabbir Khanzada | Shaukat Khanum Memorial Cancer Hospital and Research Centre | Pakistan |
| Εvgenia Μela | Hippocration General Hospital | Greece |

# **Electronic Supplementary Material - Table S1. Structured survey questionnaire.**

| **Number** | **Survey Question** |
| --- | --- |
| **Section 1 - Respondent Demographics and Practice Profile** | |
| 1 | In which country do you primarily practise? |
| 2 | What is your primary specialty? |
| 3 | What is your current position? |
| 4 | How many years have you been independently performing oesophagectomies as the primary surgeon? |
| 5 | Approximately how many oesophagectomies do you personally perform per year on average? |
| 6 | What is the approximate annual oesophagectomy volume at your institution? |
| 7 | What is your predominant operative technique for oesophagectomy? |
| 8 | What is the predominant operative platform/access used for the technique above? |
| 9 | What type of centre best describes your primary institution? |
| **Section 2 - Conduit Construction and Technical Considerations** | |
| 10 | What conduit do you most commonly fashion for oesophageal reconstruction? |
| 11 | Do you measure or formally record the diameter of the fashioned conduit? |
| 12 | What route do you use for conduit placement? |
| 13 | What is your usual level of anastomosis? |
| 14 | Do you take specific intraoperative technical steps to prevent conduit redundancy? |
| 15 | Do you place a feeding jejunostomy at the time of oesophagectomy? |
| 16 | Do you perform a cruroplasty at the time of oesophagectomy? |
| 17 | Do you fully kocherise the duodenum at the time of oesophagectomy? |
| **Section 3 - Nasogastric Tube Management** | |
| 18 | Do you routinely insert a nasogastric tube at the time of oesophagectomy? |
| 19 | What is your reason for using a post-operative nasogastric tube? |
| 20 | What is your criterion for removal of the nasogastric tube? |
| 21 | On what post-operative day is the nasogastric tube typically removed in uncomplicated cases at your institution? |
| 22 | If you use volume-based criteria for nasogastric tube removal, what 24-hour output threshold do you use? |
| **Section 4 - Early Post-Operative Feeding and Nutritional Protocols** | |
| 23 | When do patients at your institution typically begin oral free fluids post-oesophagectomy? |
| 24 | Do you perform a contrast swallow study before initiating oral intake? |
| 25 | If you perform a routine contrast swallow, on what post-operative day is this typically performed? |
| 26 | What nutritional support do you use in the immediate post-operative period while oral intake is being established? |
| **Section 5 - Incidence and Recognition of Early DGCE** | |
| 27 | Do you routinely document early DGCE as a specific complication in your institutional or national audit dataset? |
| 28 | In your experience, what proportion of your oesophagectomy patients develop early DGCE? |
| 29 | What is your estimate of early DGCE incidence based on? |
| 30 | In your clinical practice, which criteria or findings trigger a diagnosis of early DGCE, defined as ≤14 days post-operative? |
| **Section 6 - Incidence and Recognition of Late DGCE** | |
| 31 | Do you routinely document late DGCE as a specific complication in your institutional or national audit dataset? |
| 32 | In your experience, what proportion of your oesophagectomy patients develop late DGCE? |
| 33 | What is your estimate of late DGCE incidence based on? |
| 34 | In your clinical practice, which criteria or findings trigger a diagnosis of late DGCE, defined as >14 days post-operative? |
| **Section 7 - Prophylactic Pyloric Strategies at the Time of Oesophagectomy** | |
| 35 | Do you perform a prophylactic pyloric drainage or intervention procedure at the time of oesophagectomy? |
| 36 | If you perform prophylactic pyloric intervention selectively, what criteria guide your decision? |
| 37 | Which prophylactic pyloric procedure do you perform most commonly? |
| 38 | If you perform prophylactic pyloric botulinum toxin injection, what dose do you typically use? |
| 39 | If you perform prophylactic pyloric balloon dilatation, what balloon diameter do you typically use? |
| 40 | What is your rationale for performing a prophylactic pyloric procedure? |
| 41 | What is your rationale for not performing a prophylactic pyloric procedure? |
| 42 | Do you believe the potential benefits of prophylactic pyloric drainage in reducing DGCE outweigh the risks of increased bile reflux and dumping syndrome? |
| **Section 8 - Therapeutic Management of Established DGCE** | |
| 43 | In the absence of a surgically modifiable anatomical or mechanical cause, such as paraconduit hernia or conduit torsion, what is your first-line management when you encounter early DGCE, defined as ≤14 days? |
| 44 | In the absence of a surgically modifiable anatomical or mechanical cause, what is your approach to early DGCE when the first-line intervention fails? |
| 45 | In the absence of a surgically modifiable anatomical or mechanical cause, which interventions do you employ for late DGCE refractory to conservative measures? |
| 46 | If you perform therapeutic pyloric botulinum toxin injection, what dose do you typically use? |
| 47 | If you perform therapeutic pyloric balloon dilatation, what balloon diameter do you typically use? |
| 48 | In what proportion of DGCE cases do you find a single endoscopic pyloric intervention achieves sustained resolution? |
| 49 | Which prokinetic agent or agents do you most commonly use for DGCE? |
| **Section 9 - Surveillance and Follow-Up Practices** | |
| 50 | How frequently do you follow up oesophagectomy patients in the first year post-operatively? |
| 51 | During routine follow-up after oesophagectomy, how is surveillance endoscopy scheduled in the first 2 years post-operatively? |
| 52 | During routine follow-up after oesophagectomy, how is surveillance CT scan scheduled in the first 2 years post-operatively? |
| 53 | During routine follow-up after oesophagectomy, how is surveillance PET scan scheduled in the first 2 years post-operatively? |
| **Section 10 - Barriers to Optimal Management and Research Gaps** | |
| 54 | What do you consider the most significant barriers to optimal DGCE management at your institution? |
| 55 | What do you consider the single most important unanswered research question regarding DGCE? |

# **Electronic Supplementary Material - Table S2. Operative practice characteristics of respondents.**

| **Variable** | **n** | **%** |
| --- | --- | --- |
| Conduit type for reconstruction *(Most common)* |  |  |
| Narrow gastric tube (Target diameter ≤ 4 cm) | 143 | 62.2 |
| Wider gastric tube (Target diameter > 4 cm) | 70 | 30.4 |
| Whole stomach (Without tubularisation) | 8 | 3.5 |
| Colonic interposition | 6 | 2.6 |
| Jejunal free flap | 2 | 0.9 |
| Other | 1 | 0.4 |
| Conduit diameter recording |  |  |
| Measured intraoperatively with a formal tool or reference | 60 | 26.1 |
| Estimated visually intraoperatively | 102 | 44.3 |
| Not formally recorded | 68 | 29.6 |
| Conduit placement route |  |  |
| Posterior mediastinal (Native oesophageal bed) | 192 | 83.5 |
| Retrosternal / Substernal | 18 | 7.8 |
| Either - Varies by case | 20 | 8.7 |
| Level of anastomosis |  |  |
| Cervical (Neck anastomosis) | 59 | 25.7 |
| Intrathoracic (Above the azygos vein) | 70 | 30.4 |
| Intrathoracic (Below the azygos vein) | 51 | 22.2 |
| Varies by tumour location and surgical approach | 50 | 21.7 |
| Steps to prevent conduit redundancy |  |  |
| Routine careful conduit length sizing to avoid excess | 74 | 32.2 |
| Routine tacking/fixation of conduit to hiatus or pleura | 40 | 17.4 |
| Routine both sizing and fixation | 36 | 15.7 |
| Addressed only if obvious intraoperatively | 41 | 17.8 |
| No specific steps taken | 39 | 17.0 |
| Feeding jejunostomy |  |  |
| Routinely in all patients | 98 | 42.6 |
| Selectively | 71 | 30.9 |
| Never | 61 | 26.5 |
| Cruroplasty (Hiatal closure) |  |  |
| Routinely in all patients | 24 | 10.4 |
| Selectively (Large hiatal opening) | 106 | 46.1 |
| Not performed | 100 | 43.5 |
| Duodenal kocherisation |  |  |
| Routinely in all patients | 82 | 35.7 |
| Selectively | 86 | 37.4 |
| Not performed | 62 | 27.0 |

# **Electronic Supplementary Material - Table S3. Estimated incidence of early and late DGCE after oesophagectomy, as reported by survey respondents.**

| **Estimated incidence band** | **Early DGCE, n (%)** | **Late DGCE, n (%)** |
| --- | --- | --- |
| <5% | 61 (26.5%) | 83 (36.1%) |
| 5-10% | 73 (31.7%) | 69 (30.0%) |
| 11-20% | 46 (20.0%) | 39 (17.0%) |
| 21-30% | 12 (5.2%) | 12 (5.2%) |
| 31-50% | 6 (2.6%) | 4 (1.7%) |
| >50% | 1 (0.4%) | 1 (0.4%) |
| Uncertain / Do not routinely track | 31 (13.5%) | 22 (9.6%) |
| **Total** | **230 (100%)** | **230 (100%)** |

# **Electronic Supplementary Material - Table S4. Documentation and perceived incidence of DGCE by surgeon and institutional case volume.**

| **Variable** | **Surgeon volume** | | | **Institution volume** | | |
| --- | --- | --- | --- | --- | --- | --- |
|  | **≤5/Yr** | **>5/Yr** | **p** | **<20/Yr** | **≥20/Yr** | **p** |
|  | **n=123** | **n-107** |  | **n=115** | **n=115** |  |
| Early DGCE - Documentation |  |  |  |  |  |  |
| Yes - Standardised definition | 21 (17.1) | 23 (21.5) | 0.186 | 22 (19.1) | 22 (19.1) | 0.433 |
| Yes - Non-standardised definition | 42 (34.1) | 45 (42.1) |  | 43 (37.4) | 44 (38.3) |  |
| No | 38 (30.9) | 29 (27.1) |  | 30 (26.1) | 37 (32.2) |  |
| No formal audit/registry | 22 (17.9) | 10 (9.3) |  | 20 (17.4) | 12 (10.4) |  |
|  |  |  |  |  |  |  |
| Early DGCE - Perceived incidence |  |  |  |  |  |  |
| <5% | 30 (24.4) | 31 (29.0) | 0.231 | 30 (26.1) | 31 (27.0) | 0.995 |
| 5-10% | 40 (32.5) | 33 (30.8) |  | 35 (30.4) | 38 (33.0) |  |
| 11-20% | 25 (20.3) | 21 (19.6) |  | 23 (20.0) | 23 (20.0) |  |
| 21-30% | 3 (2.4) | 9 (8.4) |  | 6 (5.2) | 6 (5.2) |  |
| 31-50% | 3 (2.4) | 3 (2.8) |  | 3 (2.6) | 3 (2.6) |  |
| >50% | 1 (0.8) | 0 (0.0) |  | 1 (0.9) | 0 (0.0) |  |
| Uncertainty/not routinely tracked | 21 (17.1) | 10 (9.3) |  | 17 (14.8) | 14 (12.2) |  |
|  |  |  |  |  |  |  |
| Late DGCE - Documentation |  |  |  |  |  |  |
| Yes - Standardised definition | 23 (18.7) | 22 (20.6) | 0.794 | 21 (18.3) | 24 (20.9) | 0.385 |
| Yes - Non-standardised definition | 47 (38.2) | 36 (33.6) |  | 44 (38.3) | 39 (33.9) |  |
| No | 31 (25.2) | 32 (29.9) |  | 27 (23.5) | 36 (31.3) |  |
| No formal audit/registry | 22 (17.9) | 17 (15.9) |  | 23 (20.0) | 16 (13.9) |  |
|  |  |  |  |  |  |  |
| Late DGCE - Perceived incidence |  |  |  |  |  |  |
| <5% | 42 (34.1) | 41 (38.3) | 0.137 | 42 (36.5) | 41 (35.7) | 0.292 |
| 5-10% | 39 (31.7) | 30 (28.0) |  | 32 (27.8) | 37 (32.2) |  |
| 11-20% | 18 (14.6) | 21 (19.6) |  | 19 (16.5) | 20 (17.4) |  |
| 21-30% | 8 (6.5) | 4 (3.7) |  | 8 (7.0) | 4 (3.5) |  |
| 31-50% | 0 (0.0) | 4 (3.7) |  | 0 (0.0) | 4 (3.5) |  |
| >50% | 1 (0.8) | 0 (0.0) |  | 1 (0.9) | 0 (0.0) |  |
| Uncertainty/not routinely tracked | 15 (12.2) | 7 (6.5) |  | 13 (11.3) | 9 (7.8) |  |
|  |  |  |  |  |  |  |

P-values compare the full response distribution between volume groups. Pearson’s chi-square test was used for DGCE documentation variables. Fisher-Freeman-Halton exact tests were used where expected cell counts were sparse.

# **Electronic Supplementary Material - Table S5. Post-hoc pairwise comparisons of prophylactic pyloric intervention by operative platform, with Bonferroni correction**

| **Variable** | **χ²** | **Raw p-value** | **Bonferroni-adjusted p-value** |
| --- | --- | --- | --- |
| Open vs Hybrid | 0.9185 | 0.6318 | 1.0000 |
| Open vs Totally MIS | 7.532 | 0.0231 | 0.1386 |
| Open vs Robotic | 5.109 | 0.0777 | 0.4662 |
| Hybrid vs Totally MIS | 10.39 | 0.0055 | 0.0330 |
| Hybrid vs Robotic | 6.905 | 0.0317 | 0.1902 |
| Totally MIS vs Robotic | 0.0463 | 0.9771 | 1.0000 |

# **Electronic Supplementary Material - Table S6. Surgeon-reported likelihood of sustained DGCE resolution after a single endoscopic pyloric intervention.**

| **Response category** | **Respondents, n** | **Percentage** |
| --- | --- | --- |
| <25%: Most patients need repeat intervention | 54 | 23.5% |
| 25-50% | 54 | 23.5% |
| 51-75% | 37 | 16.1% |
| >75%: Most resolved with a single intervention | 22 | 9.6% |
| Uncertain | 63 | 27.4% |
| **Total** | **230** | **100%** |

# **Electronic Supplementary Material - Table S7. Surgeon-reported barriers to optimal DGCE management.**

| **Barrier** | **Respondents, n** | **Percentage** |
| --- | --- | --- |
| Absence of a standardised definition for DGCE | 142 | 61.7% |
| Lack of high-quality randomised evidence to guide management | 126 | 54.8% |
| Lack of DGCE-specific clinical practice guidelines | 87 | 37.8% |
| Limited access to diagnostic tools | 74 | 32.2% |
| DGCE not perceived as a priority relative to other oesophagectomy complications | 66 | 28.7% |
| Limited access to therapeutic endoscopy expertise | 61 | 26.5% |
| Lack of training or awareness of emerging DGCE management options | 40 | 17.4% |
| Hospital resource constraints limiting investigation or intervention | 32 | 13.9% |
| Insufficient or incomplete follow-up for late DGCE detection | 30 | 13.0% |
| Uncertain | 8 | 3.5% |
| No significant barrier perceived | 3 | 1.3% |

# **Electronic Supplementary Material - Table S8. Thematic analysis of surgeon-nominated research priorities for delayed gastric conduit emptying.**

| **Theme** | **Respondents (n)** | **Representative Free-Text Responses** |
| --- | --- | --- |
| Prevention strategy and intraoperative technique | 20 | *"What is the optimal, evidence-based prevention strategy for DGCE after oesophagectomy that improves long-term functional outcomes without increasing adverse effects?"*  *"Do prophylactic pyloric drainage procedures reduce the incidence of DGCE?"*  *"Is vagus nerve preservation an option to avoid this problem?"* |
| Standardised definition, diagnostic criteria, and severity grading | 19 | *"A simple consensus definition and grading that is easy to apply in daily clinical management"*  *"Establishment of a standardised, consensus-based diagnostic criteria and severity grading for DGCE"*  *"Severity threshold to define DGCE, as all oesophagectomy patients develop DGCE to a certain extent"* |
| Optimal treatment and management strategy | 18 | *"What is the most effective, standardised treatment strategy (medical vs endoscopic vs combined) to prevent and manage DGCE, and which patients benefit most from each intervention?"*  *"The best management option for early DGCE"*  *"When is it appropriate to consider failure of conservative measures for early DGCE?"* |
| Evidence gaps, trials, and clinical guidelines | 13 | *"Lack of high-quality evidence (RCTs) to guide management because of absent proper trials"*  *"Lack of clinical practice guidelines specific to DGCE prevention and management"*  *"Resource constraints - lack of funding limiting investigation or intervention"* |
| Patient selection for pyloric intervention | 10 | *"Which patients truly benefit from prophylactic or early pyloric intervention after oesophagectomy, and what standardised pathway best prevents and treats DGCE"*  *"No real-time tool/test to tell us which specific patient actually needs a drainage procedure and which patient’s pylorus will function normally without one"*  *"Determining the optimal balance between prophylactic pyloric drainage and long-term functional quality of life"* |
| Incidence, burden, and quality-of-life impact | 6 | *"Which is the impact of DGCE on patients’ quality of life?"*  *"Frequency and consequence of DGCE"*  *"If we perceive DGCE as an actual complication after surgery that needs proper evaluation"* |
| Pathophysiology, risk factors, and predictors | 5 | *"Identifying the main pathophysiological mechanism behind DGCE and predicting which patients will develop it and which won’t"*  *"What are the risk factors (anatomical [conduit morphology, blood supply of right gastro-epiploic], comorbidities, surgical) for DGCE?"*  *"Why do some patients get it and others do not when operative technique remains standard?"* |
| Diagnostic pathways and key investigations | 3 | *"The approach to investigating DGCE - the vast majority of late ‘functional’ DGCE is related to anatomical and structural problems with the conduit, hence why pyloric interventions don’t necessarily alter the outcome"*  *"Single most important diagnostic tool for DGCE"*  *"What is the cause of persistent nausea despite no radiological and endoscopic evidence of DGCE?"* |
